# A Novel On-Wrist Fall Detection System Using Supervised Dictionary Learning Technique

**DOI:** 10.1007/978-3-030-51517-1_15

**Published:** 2020-05-31

**Authors:** Farah Othmen, Mouna Baklouti, André Eugenio Lazzaretti, Marwa Jmal, Mohamed Abid

**Affiliations:** 8 Digital Research Centre of Sfax, Sfax, Tunisia; 9 Institut Mines-Télécom, CNRS, Paris, France; 10 Université de Sherbrooke, Sherbrooke, QC Canada; 11 Digital Research Centre of Sfax, Sfax, Tunisia; 12 University of Sfax, Sfax, Tunisia; 13 Ecole Polytechnique de Tunisie, Universite de Carthage, La Marsa, Tunisia; 14 Telnet Innovation Labs, Telnet Holding, Ariana, Tunisia; 15 CES Lab, National School of Engineers of Sfax, University of Sfax, Sfax, Tunisia; 16 Federal University of Technology, Paraná (UTFPR), Curitiba, Brazil

**Keywords:** Fall detection, Supervised Dictionary Learning, Machine learning, Wrist-based wearable, Signal processing

## Abstract

Wrist-based fall detection system provides a very comfortable and multi-modal healthcare solution, especially for elderly risking falls. However, the wrist location presents a very challenging and unstable spot to distinguish falls among other daily activities. In this paper, we propose a Supervised Dictionary Learning approach for wrist-based fall detection. Three Dictionary learning algorithms for classification are invoked in this study, namely SRC, FDDL, and LRSDL. To extract the best descriptive representation of the signal data we followed different preprocessing scenarios based on accelerometer, gyroscope, and magnetometer. A considerable overall performance was obtained by the SRC algorithms reaching respectively 99.8%, 100%, and 96.6% of accuracy, sensitivity, and specificity using raw data provided by a triaxial accelerometer, accordingly overthrowing previously proposed methods for wrist placement.

## Introduction

The elderly population rate has witnessed dramatic growth over the last decades and is projected to be still increasing throughout the upcoming years to reach 35% by the year 2050, and thus, jointly increasing the population dependency rate [17]. Falling is one of the most crucial health risks faced by this fragile population, classified as a disease in the International Classification of Diseases [27]. According to [16], the risk of falling varies from 30% for elderly over 65 to 50% for those over 85 each year.

Wearable fall detection systems have captivated much interest in later years literature as they can fit easily into smart wearable accessories like wristbands assuring anywhere-anytime accessibility and comfortable use compared to other existing solutions, i.e the vision and ambient-based [22]. Commonly, state-of-the-art methods for wearable fall detectors are either threshold-based or machine learning-based, for which the latter received superior interest recently [29]. Abstracting an optimal combination between extracted features and classifiers, while enhancing system reliability, has been extensively researched in most related works [19, 23]. However, classification performance can degrade substantially, as hand-crafted features may be very specific to the sensor, device placement, or dataset [2, 8].

Dictionary learning approaches (DLA) have gained a lot of enthusiasm in image processing including sparse representation based classification algorithm for face recognition [30], as it has shown robustness especially for a limited number of channels and samples, thus reducing the need to select the best feature combination and classifier for the application. Therefore, DLA has been recently emerged into the biomedical signal processing field, of which some associated works have been proposed mainly for Electroencephalography (EEG) and electrocardiogram (ECG) signal classification [3, 13].

In the same direction, we propose in this paper a novel on-wrist fall detection system based on Supervised Dictionary Learning (SDL), to autonomously generate optimal features selection that best represents acquired data. Indeed, the work presented here extends previous study [19] that implemented a movement decomposition method to extract features (direction components and body orientation) and machine learning algorithms for fall detection based on wrist wearable device. For evaluation purposes, three SDL and sparse representations algorithms with different experimental situations will be assessed throughout this paper, besides comparing it with previous related works. In this context, multiple sensors and features combinations in different experimental arrangements will be used. To the extent of our knowledge, such a dictionary-based approach is still underexplored in the related literature, so it is the main contribution of this work.

The remainder of this paper is organized as follows. Section 2 presents the main theoretical background behind our study. A detailed description of our proposed method is provided in Sect. 3. Section 4 illustrates the obtained results and compares them with prior works. Conclusion and future related work are provided in Sect. 5.

## Theoretical Background and Related Work

### Wearable Fall Detection System

Wearable-based fall detection systems illustrate all on-body attached garment devices that usually embed inertial measurement units (IMU) to inspect the body’s motions, positions, and rotation movements in the space [22]. Commonly, inertial sensors such as accelerometers, gyroscopes, and magnetometers are the most used for fall detection to discriminate and notify the occurrence of a fall event as soon as possible [15]. It mostly presents an ideal solution for indoor and outdoor monitoring, especially with the emergence of nowadays advances in wearable technologies like a pendant, band, and glasses to make it more comfortable and tolerable to be wear.

Most of the analysis methods being employed in wearable fall detection are grounded on threshold and machine learning algorithms [10]. Threshold-based approaches usually compare the sensor’s acquired data (or extracted features) with a predefined threshold(s) and a fall is detected when the predefined value is exceeded [26]. However, these algorithms are practically unreliable as fall is often confused with other activities like jumping. Additionally, a huge amount of soft falls are likely to be unidentified, due to their low threshold [6]. To enhance the accuracy limitation of the threshold algorithms, the literature proposed various machine learning-based solutions through classification algorithms like SVM, ANN, KNN, etc [1, 23]. These algorithms are more efficient as they can globe a greater number of fall types, yet very dependent of the on-body placement. Thus, machine-learning algorithms have shown impressive practical results when placed in steady body location (near gravity point of the body) such as waist and chest-worn. Otherwise, they are less efficient especially when placed in extremities such as wrist, requiring further investigations to improve the performance in those cases, mainly because wrist-based solutions are the most comfortable from a user point of view and less associated to the stigma of using a medical device [12, 18, 19].

### Dictionary Learning for Classification

**Sparse Representation and Supervised Dictionary Learning Characteristics.** DLA has received a lot of interest as a representation learning paradigm by achieving state-of-the-art performance in many practical fields in computer vision such as information retrieval, image restoration, and classification [5].

It has been observed that DLA intends to learn a dictionary directly from the training samples by generating the space where the given signal could be represented properly to provide improved processing and better results in fitted to the problem domain. In DLA models, given a set **X** = [$\mathbf{x} _1$,... , $\mathbf{x} _m$] of m samples, the objective is to generate a dictionary **D** which maps a high and sparse dimensional representation denoted **A** for each input sample. Generally, one can obtain this by solving an optimization problem defined by the following equation:

1 $$\begin{aligned} \min _\mathbf{D ,\mathbf{A} }\sum _{i=1}^{m}(\frac{1}{2}||\mathbf{x} _i - \mathbf{D} {} \mathbf{a} _i||_2^2 + \lambda _1||\mathbf{a} _i||_1), \end{aligned}$$

where, $\lambda _1$ defines the regularization parameter that affects the number of nonzero coefficients.

To cover classification tasks, many techniques have been proposed in the literature [5]. The latter, exploit the label information in the learning of either the dictionary atoms, the coefficients of the sparse vector, or both. Based on [21], both extra restraint function $f_{A}(.)$ and $f_{D}(.)$ are added to Eq. (1) that satisfies:

2 $$\begin{aligned} \min _\mathbf{D ,\mathbf{A} }\{\sum _{i=1}^{m}(\frac{1}{2}||\mathbf{x} _i - \mathbf{D} {} \mathbf{a} _i||_2^2 + \lambda _1||\mathbf{a} _i||_q) + \lambda _2 f_{A}(\mathbf{A} ) + \lambda _3f_D(\mathbf{D} )\}, \end{aligned}$$

where, $f_{A}(.)$ could be a logistic function, a linear classifier, a label consistency term, a low-rank constraint, or the Fisher discrimination criterion. As for $f_{D}(.)$ is to force the incoherence of the dictionary for different classes. Hence, it is possible to jointly learn the dictionary and classification model, which attempt to optimize the learned dictionary for classification tasks [9]. $\lambda _2$ and $\lambda _3$ are two scalar parameter corresponding respectively to the associated function [5].

Assuming that SDL methods and sparse representation differ in the way they exploit class labels, we will detail three of the most popular SDL algorithms, namely, the SRC, FDDL, and LRSDL.

**Sparse Representation-Based Classification (SRC).** SRC was first proposed by Wright et al. in their work [28] with robust face recognition approach, and have accordingly proved its effectiveness for low to moderate amount of data based problems [5]. This approach aims to concatenate the training data from different classes into a single dictionary and uses class-specific residue for the recognition. Thus, the test samples are represented as a linear combination of just the training samples corresponding to the same class. Literally, no actual training is performed in his method, since the integrity of the training samples are used in the dictionary and the sparse representation is extracted and classified over the testing phase following two main stage process:

1. The SRC algorithm computes the sparse coefficient $\mathbf{a}$ of the test sample $\mathbf{x} _{test}$ via the *Lasso* equation as:
  3 $$\begin{aligned} \min _\mathbf{a }\{\frac{1}{2}||\mathbf{x} _{test} -\mathbf{D} {} \mathbf{a} ||_2^2 + \lambda _1||\mathbf{a} ||_1\}, \end{aligned}$$
  Assuming that $$\mathbf{D} = \mathbf{X} _{train} $$.
2. Class label of each test sample is assigned while maintaining a minimum residual error of the classes according to:
  4 $$\begin{aligned} Label(\mathbf{x} _{test})= \min _i r_i(\mathbf{x} _{test}), \end{aligned}$$
  where, $r_i = ||\mathbf{x} _{test} - \mathbf{D} \sigma _i(\mathbf{a} )||_2^2$, $\sigma _i$ is the selective function of the coefficient vector associated to the class *i*.

**Fisher Discrimination Dictionary Learning (FDDL).** In [31], Yang et al. proposed an SDL method that learns class-specific structured dictionary while managing its discriminability through adding a Fisher criterion. Thus, the learned dictionary $\mathbf{D} = [\mathbf{D} _1,\mathbf{D} _2,..,\mathbf{D} _m]$, where $\mathbf{D} _i$ is a sub-dictionary corresponding to the class *i*, powerfully represents the inter-class similarity and the intra-class variance. To describe FDDL more formally, suppose $\mathbf{X} =[\mathbf{X} _1,\mathbf{X} _2,..,\mathbf{X} _c ]$, such as the training samples are grouped according to the classes they belong and c is the total number of classes. The overall objective function of FDDL is written as shown by Eq. (5):

5 $$\begin{aligned} \min _\mathbf{D ,\mathbf{A} }\{ r(\mathbf{X} ,\mathbf{D} ,\mathbf{A} )+\lambda _1||\mathbf{A} ||_1+\lambda _2f(\mathbf{A} )\}, \end{aligned}$$

where, $\mathbf{A} =[\mathbf{A} _1,\mathbf{A} _2,..,\mathbf{A} _c]$ regroups the sparse representation of each training sample over **D**; $r(\mathbf{X} ,\mathbf{D} ,\mathbf{A} )$ is the Fisher fidelity term; $f(\mathbf{A} )$ defines the discrimination constraint.

### Low-Rank Shared Dictionary (LRSDL)

Vu et al. proposed an SDL framework in their works [24, 25], that aims to enhance the capability of capturing shared features of the FDDL approach. The LRSDL approach intent to simultaneously learn sub-dictionaries with discriminative and shared features of each class, as different classes often share common patterns. Accordingly, the main focus of the LRSDL is the shared part in which two intuitive constraints are added to the corresponding objective function. The first one is the low-rank structure constraint, that allows the shared dictionary to contain some discriminative features. As for the second, the sparse coefficients corresponding to the shared dictionary should be very similar.

## Proposed Dictionary Learning Method

Considering that the wrist-worn devices are the most comfortable body location for the patient [18], they are yet very unstable for the IMU [32]. Since arms are usually very moving parts of the body, many hand movements, i.e clapping, rising, and releasing hands, may present similar motion patterns compared with fall movements. Thus, these movement similarities may present a bottleneck for the feature extraction task as it may become very specific to the collected data and the selected sensors.

**Fig. 1.**
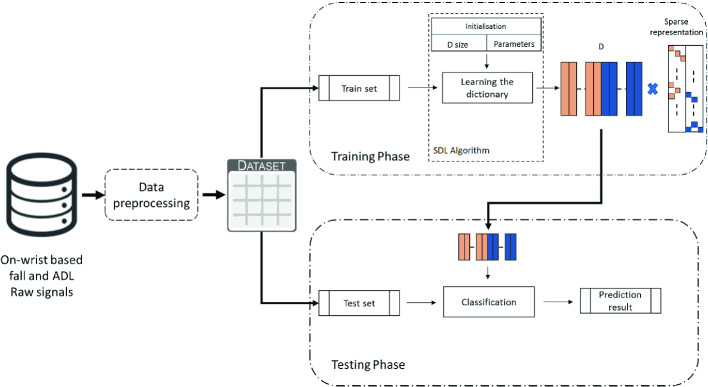
Pipeline overview of the proposed SDL-based fall detection system.

To overcome this issue while bearing in mind the system reliability, we propose a fall detection approach based on the dictionary learning algorithms for classification. Therefore, different SDL classification algorithms will be evaluated and compared through their prediction performances with previous on-wrist solutions presented in the literature. The pipeline of the designed architecture is illustrated by Fig. 1. In this section, we will describe the main phases presented in the illustration, namely the preprocessing, the training, and the test phases.

### Dataset

The data set has been collected throughout de Quadros et al. study [19]. In fact, the signal acquisition was done by the use of three main triaxial IMU sensors, i.e, accelerometer, gyroscope, and magnetometer which are embedded in the GY-80 IMU model device. To acquire and register data signals from the latter sensors, an Arduino Uno was integrated with the IMU device into a wrist-worn band at the non-dominant hand. The raw sensors data were obtained in a 100 Hz sampling rate and 4 g, 500 degrees/sec, and 0.88 Gs for the accelerometer, gyroscope, and magnetometer respectively.

In order to make the data set more generalized and accurate, twenty-two volunteers with different ages, heights, and weights were engaged in this experimental protocol. Each one performs two main event categories, namely, fall incidents and Activities of Daily Living (ADL). The recorded fall incident covers forward to fall, backward fall, right-side fall, left-side fall, fall after rotating the waist clockwise, and fall after rotating the waist counterclockwise. The ADL’s performed activities enclose walking, clapping hands, moving an object, tying shoes and sitting on a chair. The average duration of the recorded activities is 9.2 s, assuming that each one starts with a resting arm (resting state) followed by a few steps before the activity’s performance.

For the sake of removing any external influence that affects the accelerometer [6], the accelerometer data was preprocessed with a low pass filter with a window size of 40 and a subtraction of a fixed value equal to 1 g to eliminate the gravity-related information.

### Data Preprocessing

Most of the proposed wearable fall detection relies, mainly, on the data preprocessing phase, including feature extraction and feature selection, as it plays a critical role in defining an accurate fall detector [14]. In this sense, one of the faced challenges for this placement is extracting relevant features that better describe raw data and discriminate ADL events from a fall event, especially for overlapped and similar data. Finding significant attributes that better illustrate the raw data has always been a challenge depending on the device’s on-body position. For instance, most on-wrist solution presented in the literature depends mainly on accelerometer [4, 11, 20, 34], while some others fuses it with other sensors like gyroscope [7, 32, 33], gyroscope and magnetometer [19], or heart rate sensor [14].

**Fig. 2.**
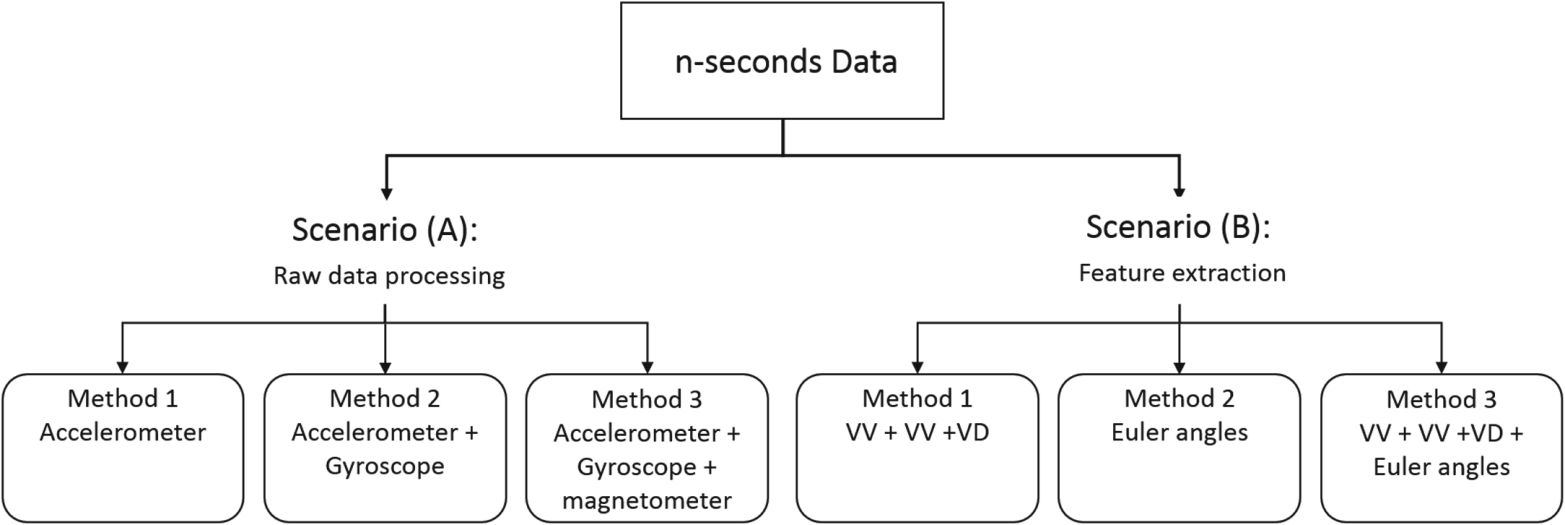
Proposed scenarios for data preprocessing.

This work implements SDL for classification approaches in a wrist-based fall detection system with the aim of benefiting of its capacity to generate more discriminative features using sparse representation. For this purpose, we consider two scenarios as demonstrated in Fig. 2. In scenario (A), the system will process a time window of raw data, where we will test the effect of each sensor in the system efficiency by adding one sensor at a time. The second scenario (B) experiments extracted features, as we will adopt the movement decomposition-based feature extraction method used in [19]. We will only acknowledge the vertical component of the movement and the orientation decomposition as it reached the best results in the latter work. We denote VA, VV, and VD respectively as Vertical Acceleration, Vertical Velocity, and Vertical Displacement. The Euler angles present the spacial orientation features, i.e. yaw, pitch, and roll.

### Dictionary Learning for Fall Detection

As being a branch of Machine Learning, the classification based on SDL involves two main phases, namely the training and the testing phases. In the training phase, the goal of the SDL algorithm is to map the low dimensional training data to a high and sparse dimensional representation using a learned dictionary **D**, to make a more discriminated pattern and easier to be distinguished. In this paper, we consider three SDL algorithms, SRC, FDDL, and LRSDL, that we previously detailed in Sect. 2.2.

Considering the test phase, the testing sample can be classified by directly coding it over the obtained **D**. Generally, the sparse code is then used as a feature descriptor of the data in order to calculate the reconstruction error associated to each class. The prediction is accorded to the class with the least error following the formula expressed by Eq. (4). However, the SDL performance is directly affected by the dictionary size. To abstract each SDL’s higher performance we will inspect the impact of the Dictionary size into the system’s accuracy.

## Experimental Validation

### Performance Metrics

This study is evaluated in terms of three common metrics, namely, Accuracy (AC), Sensitivity (SE), and Specificity (SP). AC represents the overall true detection, SE represents the ability to detect authentic falls among all detected falls, and SP represents the capacity to detect real ADL in all the detected ADL.

### Experimental Configuration

In our experimental analysis, we assume that a 4-s time window is sufficient to extract a fall or an ADL event. We consider that the collected data set is subdivided such as 75% of the data (nearly 300 samples for each class) is for the training phase and 25% for the test phase. From our experiments, the SDL algorithms’ hyper-parameters are set based on the best-achieved performances for our dataset using random training features. Thus, we initiate them as follows: SRC: $\lambda =0.01$; FDDL: $\lambda _1 = \lambda _2=0.001$; LRSDL: $\lambda _1=0.001$, $\lambda _2=0.01$, $\eta =0.02$. Throughout this study, the size of the dictionary **D** for the FDDL and the LRSDL algorithm will vary between 50 and 300 atoms per class depending on the experiment.

### Experimental Result

In this study, we followed two main experimentation scheme to validate the high sensitivity and efficiency of our proposed method. Firstly, we fix the Dictionary size in order to assess the SDL classification performance behavior compared with each outline of both scenarios showcased in Fig. 2. Secondly, we evaluate the best performance of the previous experiment with multiple **D** sizes for the FDDL and LRSDL algorithms to exhibit for each the best-fitted size to our proposed system.

**Table 1.** Performance comparison for different methods of raw data scenario.

Algorithm	SRC			FDDL			LRSDL		
	AC	SE	SP	AC	SE	SP	AC	SE	SP
Acc	**99.8**	100	99.6	98.0	98.0	98.0	97.4	97.9	96.9
Acc, Gyr	**90.6**	90.6	90.6	**90.6**	93.8	87.5	90.1	93.8	86.5
Acc, Gyr, Mag	**97.4**	96.9	97.9	96.4	96.9	95.8	**97.4**	96.9	97.9

**Table 2.** Performance comparison for different methods of feature extraction scenario.

Algorithm	SRC			FDDL			LRSDL		
	AC	SE	SP	AC	SE	SP	AC	SE	SP
VA, VV, VD	**96.4**	97.9	94.8	95.9	99.0	92.7	95.8	99.0	92.7
Euler	**99.5**	99.0	100	98.4	100	96.9	98.4	100	96.88
(+Euler)	**96.9**	96.9	96.9	95.8	96.9	94.8	96.4	95.8	96.9

**1st Experiment.** The SRC algorithm generates a dictionary **D** with the size of the training samples, we set a **D** size of 300 atoms per class.

Table 1 and Table 2 exhibit respectively the performance of the tested SDL algorithms under Scenario (A) and Scenario (B). In Table 1, an impressive performance is achieved by the SRC algorithm using a single triaxial accelerometer raw data. Even though joining the gyroscope has significantly decreased efficiency, it has proved its convenience when fused with the magnetometer. Table 2 shows that the extracted spacial orientation angles present a better accuracy compared with it when fused with a vertical movement component. Overall, the SRC has reached the best accuracy of 99.8% compared to FDDL and LRSDL when processed with a raw data accelerometer.

**Fig. 3.**
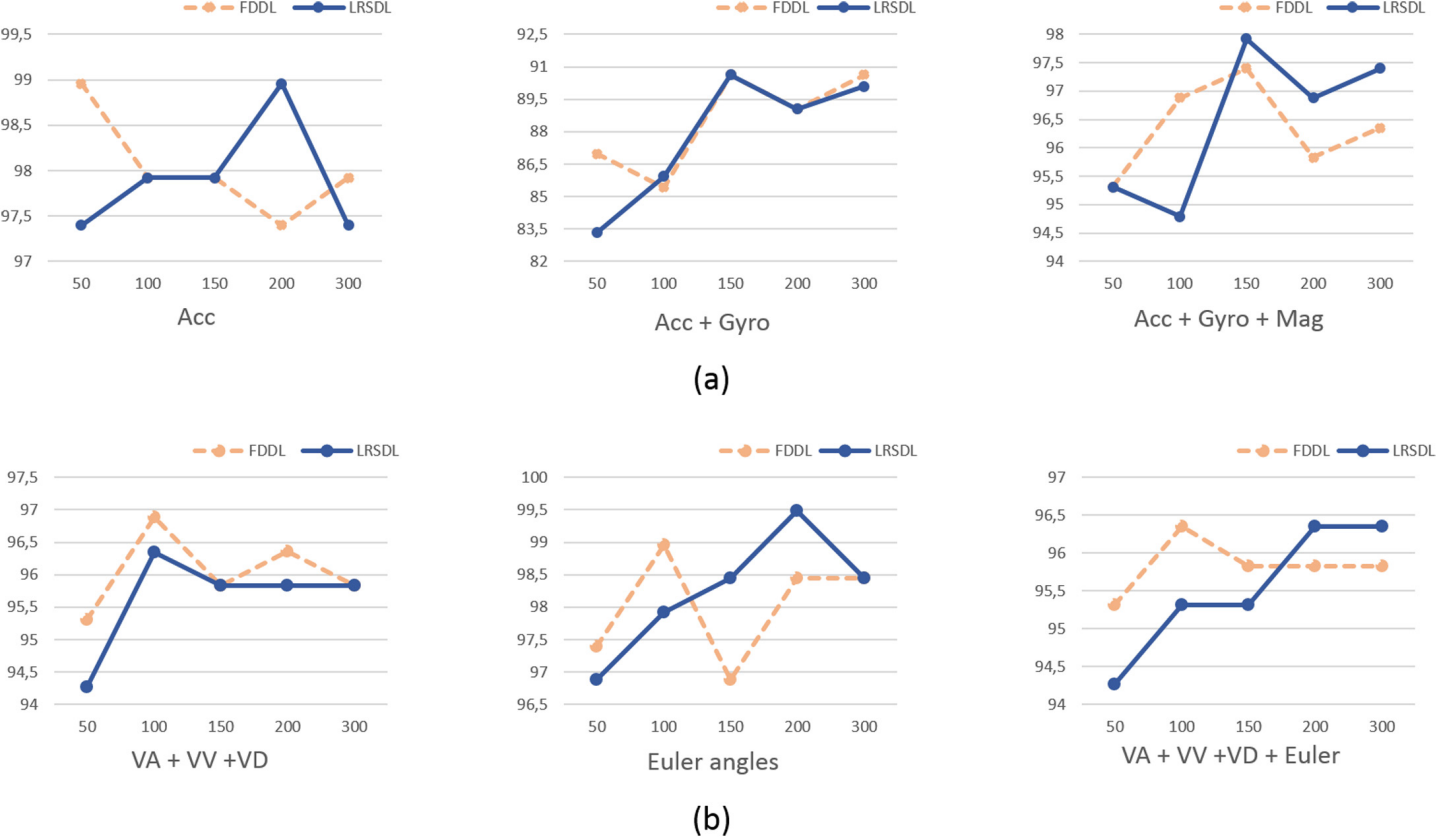
Performance of the FDDL and LRSDL for different D size, (a) Scenario A, (b) Scenario B.

**2nd Experiment.** In order to inspect the best performance of both SDL algorithms, i.e FDDL and LRSDL, we vary the **D** size in the range of $[50, \dots , 300]$ atoms per class. As illustrated in Fig. 3, the change in SDL performance depends roughly on the patterns of the input set. Consequently, the LRSDL has reached the best accuracy of 99.5%, when processed with Euler angles an input data and **D** presents a total of 400 atoms.

**Table 3.** Comparison of performance for related on-wrist fall detectors.

	[34], 2014	[7], 2014	[14], 2016	[19], 2018	[11], 2018	[20], 2019	[32], 2019	[33], 2019	Our work
Accuracy	93.75	NA	92.9	99.0	95.47	98.1	98.36	99.86	**99.8**
Sensitivity	83.33	95.0	80.95	100	83.33	98.1	95.1	99.93	**100**
Specificity	95.4	96.7	98.35	97.9	95.96	98.1	100	99.8	**99.6**

We listed in Table 3 a full synthesis of performances, in terms of sensitivity, specificity, and accuracy of prior works related to the on-wrist fall detection system. Zheng et al. [33] achieved the best accuracy performance of 99.86% with the use of an accelerometer and gyroscope using the Convolution Neural Network (CNN) architecture, yet very close with the one accomplished with our proposed study using a single sensor adopting a simpler algorithm SRC. Moreover, our work reached the maximum sensitivity of 100% likewise the one obtained by de Quadros et al. [19] resulting in a maximum ability to distinguish real falls, thereafter a more reliable system.

## Conclusion

In this work, we introduced a new classification method, Dictionary Learning, for a wrist-based fall detection system. Thus, our contribution mainly lies in applying the Supervised Dictionary Learning approach into an on-wrist fall detection system as it has not been explored yet in literature. We explored three main SDL algorithms, namely SRC, FDDL, and LRSDL with different experiments in order to abstract the best performer and compares it to those reported in previous related work. The SRC has proved its efficiency reaching respectively 99.8%, 100%, and 96.6% of accuracy, sensitivity, and specificity. Indeed, our proposed method has proven the best capacity to classify real falls correctly and the higher accuracy with just one accelerometer mounted. This solution is energy efficient compared with the one presenting similar accuracy thanks to its simpler algorithm complexity compared with the CNN architecture.

Thorough experimentation will be conducted in future work, we expect an additional improvement of results even further. As being a popular representation based paradigm, we plan next to test the performance of the SDL on jointly learn a frame-like representation of further complex patterns like cepstral representations and classification parameters in order to enhance the system’s reliability.

In our future related work, we will study a further advantage of the DLA benefits by testing its robustness in regards to noisy signals. For this proposal, we will be combining a Signal-to-Noise Ratio (SNR) to the raw signal and compare its performance behavior with traditional machine learning models.
